# Reticulocyte binding protein homologues are key adhesins during erythrocyte invasion by *Plasmodium falciparum*

**DOI:** 10.1111/j.1462-5822.2009.01358.x

**Published:** 2009-07-30

**Authors:** Tony Triglia, Wai-Hong Tham, Anthony Hodder, Alan F Cowman

**Affiliations:** The Walter and Eliza Hall Institute of Medical ResearchMelbourne 3050, Australia

## Abstract

The Apicomplexan parasite responsible for the most virulent form of malaria, *Plasmodium falciparum*, invades human erythrocytes through multiple ligand–receptor interactions. The *P. falciparum* reticulocyte-binding protein homologue (PfRh or PfRBL) family have been implicated in the invasion process but their exact role is unknown. PfRh1 and PfRh4, members of this protein family, bind to red blood cells and function in merozoite invasion during which they undergo a series of proteolytic cleavage events before and during entry into the host cell. The ectodomain of PfRh1 and PfRh4 are processed to produce fragments consistent with cleavage in the transmembrane domain and released into the supernatant, at about the time of invasion, in a manner consistent with rhomboid protease cleavage. Processing of both PfRh1 and PfRh4, and by extrapolation all membrane-bound members of this protein family, is important for function and release of these proteins on the merozoite surface and they along with EBA-175 are important components of the tight junction, the transient structure that links the erythrocyte via receptor–ligand interactions to the actin–myosin motor in the invading merozoite.

## Introduction

*Plasmodium falciparum* causes the most severe form of malaria in humans and results in approximately 500 million infections and over two million deaths each year (Snow *et al*., 2005). The asexual blood stage of the parasite life cycle multiplies rapidly within the erythrocytes and is responsible for the disease manifestations of malaria. The invasive merozoite forms are released from mature schizont stages and these rapidly invade new red blood cells in a process mediated by a cascade of events that includes multiple receptor–ligand interactions (see for review Cowman and Crabb, 2006). The merozoite has, at the apical end, specialized organelles known as rhoptries and micronemes that are responsible for storage and release of the ligands required for host cell invasion. After initial interaction of the merozoite with the erythrocyte reorientation occurs and a tight junction is formed with the host cell by an array of receptors with parasite ligands that are ultimately linked to an actin–myosin motor. The tight junction is propelled progressively along the surface of the invading merozoite by virtue of the force generated by the motor until membrane fusion at the posterior end of the parasite resulting in internalization within a parasitophorous vacuole. This invasion process is tightly controlled and completed within approximately 20–30 s.

The different steps in merozoite invasion appear to involve different ligand–receptor interactions (see for review Gaur *et al*., 2004; Cowman and Crabb, 2006). Initial binding of the merozoite is via low affinity interactions whereas tight junction formation at the merozoite–erythrocyte interface appears to require high affinity interactions that in *P. falciparum* probably involve two protein families known to be important in recognition and invasion of the human erythrocyte. The first family includes the microneme-located *P. falciparum* erythrocyte binding-like (ebl) proteins consisting of EBA-175, EBA181 (also known as JESEBL) and EBA140 (also known as BAEBL) (Adams *et al*., 1990; 1992; 2001). These proteins are members of the Duffy binding-like family that includes Duffy binding orthologues in *Plasmodium vivax* and *Plasmodium knowlesi* (Adams *et al*., 1990). EBA-175 functions in invasion by binding to glycophorin A (Adams *et al*., 1990) while the orthologue EBA140 binds to glycophorin C (Mayer *et al*., 2002; Maier *et al*., 2003; Lobo *et al*., 2003).

The second family shown to play an important role in merozoite invasion is the reticulocyte binding-like proteins and includes *P. vivax* reticulocyte binding proteins 1 and 2 (PvRBP-1 and -2) (Galinski *et al*., 1992) and the Py235 family in *Plasmodium yoelii* (Preiser *et al*., 2002). In *P. falciparum* this family includes PfRh1, PfRh2a, PfRh2b, PfRh4 and PfRh5 and these proteins are localized at the neck of the rhoptries in the merozoite (Rayner *et al*., 2000; 2001; Triglia *et al*., 2001; 2005; Kaneko *et al*., 2002; Duraisingh *et al*., 2003). The gene potentially encoding a sixth protein, PfRh3, appears to be a pseudogene at least in the strains that have been analysed to date (Taylor *et al*., 2001). PfRh1 binds to erythrocytes in a sialic acid-dependent manner as this interaction is sensitive to neuraminidase-treatment of the host cell and the properties of the receptor have been defined although it has yet to be identified and is currently called ‘Y’ (Rayner *et al*., 2001; Gao *et al*., 2008). Binding of PfRh4 to erythrocytes is neuraminidase-resistant and similarly its erythrocyte receptor ‘W’ is yet to be identified (Stubbs *et al*., 2005); however, a domain of PfRh4 has been shown to encode the binding region of this 205 kDa protein (Gaur *et al*., 2007; Tham *et al*., 2009). PfRh2a and PfRh2b have not been shown to bind erythrocytes (Duraisingh *et al*., 2003).

In contrast to other members of Apicomplexa, such as *Toxoplasma gondii* with a wide host range, *Plasmodium* species have a very narrow range of cell types that can be invaded within the blood stream. Despite this restricted host cell specificity *P. falciparum* can utilize different patterns of multiple ligand–receptor interactions thus providing a mechanism of phenotypic variation to evade host immune responses and the polymorphic nature of the erythrocyte in human populations (Duraisingh *et al*., 2003). The PfRh family plays a key role in both recognition and invasion of the host erythrocyte and the ability of different parasite strains to utilize alternate receptors. This mechanism of phenotypic variation is mediated by differential expression of the PfRh proteins and activation of specific ligands. While all strains of *P. falciparum* appear to express PfRh1, the level can vary dramatically as a result of gene amplification (Triglia *et al*., 2005). Disruption of PfRh1 expression resulted in a parasite line that utilized alternate ligands as evidenced by the fact that merozoite invasion was less sialic acid-dependent (Triglia *et al*., 2005). In contrast, most strains have the *PfRh2a* and *PfRh2b* genes; however, they do not all express the proteins (Duraisingh *et al*., 2003). PfRh2b plays an important role in invasion through an alternative pathway and the properties of the erythrocyte receptor involved have been defined. Similarly while all strains analysed appear to encode the *PfRh4* gene, not all express the protein (Stubbs *et al*., 2005). Indeed some are able to activate the *PfRh4* gene resulting in a switch in receptor utilization for merozoite invasion (Stubbs *et al*., 2005; Gaur *et al*., 2006). Differential expression and activation of PfRh proteins provide the parasite with a mechanism to vary the major erythrocyte ligands used by the merozoite for invasion thus providing an important mechanism for evasion of host immune responses and receptor polymorphism (Duraisingh *et al*., 2003).

Fragments of both the ebl and PfRh protein families accumulate in the parasite supernatant suggesting they are cleaved before and/or during merozoite invasion. Indeed EBA-175 has been shown to be cleaved by *P. falciparum* rhomboid 4 (PfROM4), a rhomboid protease, within the transmembrane domain during parasite invasion resulting in the shedding of the ectodomain into the supernatant (O'Donnell *et al*., 2006). Also it has been shown in a heterologous system that PfROM4 can cleave a number of merozoite proteins including members of the PfRh family (Baker *et al*., 2006) suggesting they may also be released into the supernatant during invasion as a result of protease cleavage. Although PfRh proteins are known to play key roles in invasion it is not clear if they are involved in the tight junction. Although it is known that members of the PfRh protein family such as PfRh1 are proteolytically processed it is also not known when these events occur during the invasion process (Triglia *et al*., 2005). In this work, we address these questions and show that PfRh1 and PfRh4 undergo a series of proteolytic cleavage events resulting in a number of fragments with the final event occurring within the transmembrane domain, most likely as a result of cleavage with PfROM4, at the point of invasion resulting in shedding of a domain into the supernatant. We also provide evidence that PfRh1 is most likely located within the tight junction and therefore this protein family plays a key role in merozoite invasion.

## Results

### PfRh1 is processed to 240 and 120 kDa fragments in schizonts

**Fig. 1:**
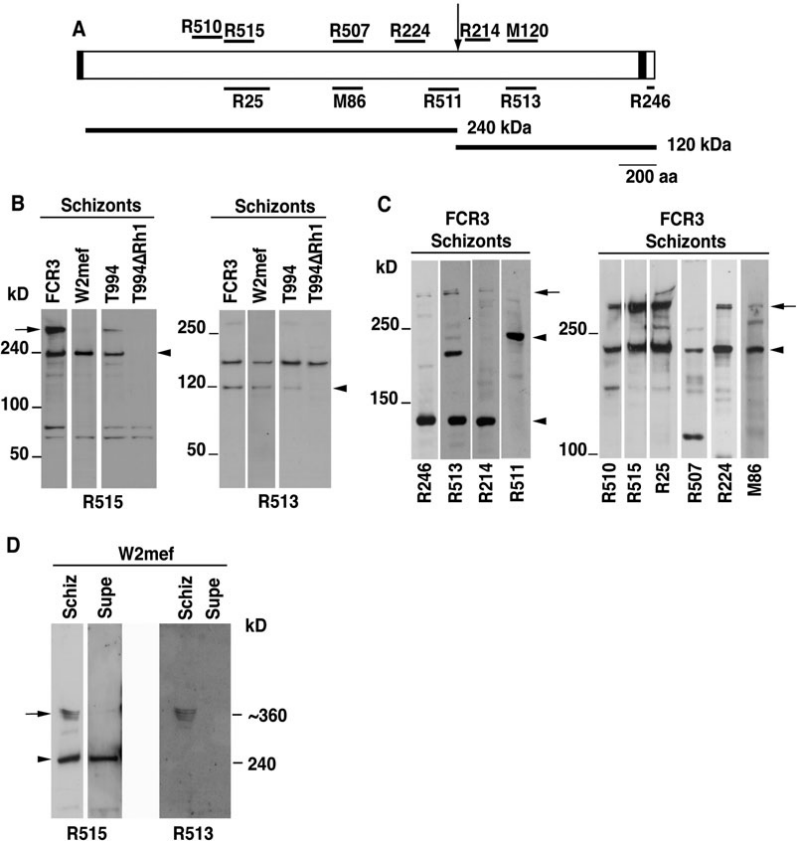
The PfRh1 protein is processed to 240 and 120 kDa products in schizonts. A. Schematic representation of the antibodies used in this study and the approximate location of the processing event leading to 240 and 120 kDa PfRh1 products. The N-terminal signal sequence and C-terminal transmembrane domain are shown. B. Antibodies detect either a 240 or 120 kDa protein in schizonts from various parasite lines. The arrowhead indicates the reactivity at 240 kDa (R515) and 120 kDa (R513) of each antibody while the arrow indicates the reactivity of the unprocessed Rh1 protein. The reactivity in the left panel between 70 and 80 kDa and the reactivity at around 180 kDa in the right panel, are both cross-reactions as they are detected even in the Rh1 knockout parasite. C. Proteins from FCR3 schizonts were probed with a panel of antibodies in order to localize the processing events in more detail. The arrowhead indicates either the 240 or 120 kDa reactivity and the arrow indicates unprocessed Rh1. The ability of different antibodies to detect the unprocessed Rh1 protein varies, so that in general antibodies to PfRh1-240 tend to recognize the unprocessed Rh1 well (left panel) while antibodies to Rh1-120 recognize it poorly (right panel). D. Proteins from either W2mef schizonts or culture supernatants were probed with R515 and R513. The arrow indicates the unprocessed PfRh1 while the arrowhead represents the 240 kDa processed fragment.

In order to determine the role of proteolytic processing for the PfRh protein family in merozoite invasion we generated a panel of 11 antibodies across the length of PfRh1 (Fig. 1A). While the *PfRh1* gene (PFD0110w) is predicted to encode a ∼360 kDa protein, only low reactivity against the full-length unprocessed PfRh1 protein was observed previously (Triglia *et al*., 2005). To detect the different processed products in schizont stages of parasite lines T994, W2mef and FCR3, we probed with an antibody (R515) to a region of PfRh1 at the N-terminus of the protein and a second near the C-terminus (R513) (Fig. 1B). To confirm the specificity of the antibodies and identify any cross-reactive bands, schizonts from a strain in which the *PfRh1* gene had been disrupted (T994ΔRh1) was also analysed (Fig. 1B) (Triglia *et al*., 2005). The R515 antibody detected a 240 kDa product (arrowhead, Fig. 1B), as well as the full-length protein > 300 kDa, while R513 detected a 120 kDa protein band (the band at approximately 160 kDa is a non-specific cross-reactive band as it is also observed in T994ΔRh1), both of which were absent in T994ΔRh1, showing that a processing event cleaves PfRh1 during schizont development. In order to localize the PfRh1 cleavage site more accurately, FCR3 schizont material was probed with antibodies made to regions along the length of PfRh1 (i.e. R246, R513, R214, R511, R510, R515, R25, R507 and R224). As the antibodies R246, R513 and R214 detected a 120 kDa band (arrowhead, Fig. 1C) while R511, R510, R515, R25, R507 and R224 detected the 240 kDa product the processing site was localized to a region between R214 and R511 (Fig. 1A and C). Therefore, PfRh1 is proteolytically processed into both 240 and 120 kDa fragments in the mature schizont stage before merozoite release.

In order to confirm that the reactivity of the PfRh1 antibodies against a large protein migrating above the 250 kDa marker was full-length PfRh1, we probed schizonts and spent culture supernatants from the W2mef parasite with antibodies to both the N- and C-termini of the protein (Fig. 1D). While both antibodies recognized the unprocessed PfRh1 at around 360 kDa, only the N-terminal antibody detected the 240 kDa processed fragment. For W2mef reactivity of the unprocessed PfRh1 varies between 5% and 20% of the reactivity against the processed fragment. However, in the FCR3 parasite that has more than 10 copies of the *PfRh1* gene, there is much more reactivity against the unprocessed form (Fig. 1C). In contrast, no unprocessed PfRh1 is found in supernatants of the W2mef parasite (Fig. 1D).

### C-terminal-tagging of PfRh1, PfRh4 and EBA-175

**Fig. 2:**
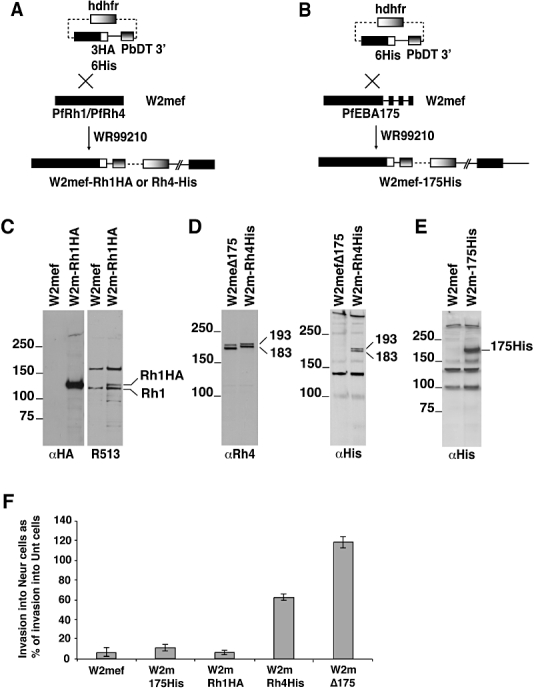
C-terminal tagging of the PfRh1, PfRh4 and EBA-175 proteins in *P. falciparum* by transfection. A. Plasmids for tagging PfRh1 and PfRh4 with either 3HA or 6His residues by 3′ cross-over recombination are shown. The human dihydrofolate reductase-thymidylate synthase (*hdhfr*) gene encodes resistance to WR99210. The whole cassette that includes a promoter and terminator is labelled as hdhfr. The *P. berghei* dhfr 3′ terminator region (PbDT-3′) served as the terminator for the gene being tagged. B. The same plasmid shown in A for adding 6His residues was used to tag the 3′ end of the EBA-175 gene (the three 3′ exons are shown) by 3′ cross-over recombination. C. PfRh1 is tagged with 3HA. Proteins from W2mef or the line expressing tagged Rh1 (W2mef-Rh1HA) were separated by SDS-PAGE then probed with either anti-HA antibodies or R513 antibodies to the 120 kDa Rh1 product. The two untagged copies and one tagged copy of *PfRh1* are labelled as PfRh1 or Rh1HA respectively. D. PfRh4 is tagged with 6His residues. Proteins from W2mefΔ175 or the line expressing tagged Rh4 (W2mef-Rh4His) were probed with anti-Rh4 or anti-His antibodies. The sizes of the tagged PfRh4 doublet are labelled as 193 and 183 kDa. E. EBA-175 is tagged with 6His residues. Proteins from W2mef or the line expressing tagged EBA-175 (W2mef-175His) were probed with anti-His antibodies. Tagged EBA-175 is labelled as 175His. F. Ability of tagged parasite lines to invade neuraminidase-treated erythrocytes. The *y*-axis shows the ratio of the invasion into treated versus invasion into untreated erythrocytes. Error bars in all cases show 95% confidence limits.

To more easily follow the processing and movement of PfRh1 and compare it with PfRh4 and EBA-175 in W2mef, we C-terminally tagged the three genes with an epitope (Fig. 2A and B). C-terminal regions of the three genes were cloned into a plasmid designed to introduce either three copies of the haemagglutinin (HA) tag or six Histidine (6His) residues by 3′ homologous cross-over recombination (Fig. 2A and B). The transfected W2mef parasites were selected to obtain lines in which the corresponding plasmids were integrated (Fig. 2A and B) and this was confirmed by probing schizont stages with antibodies to the relevant epitope tag (Fig. 2C–E). Anti-HA antibodies detected a protein of 125 kDa in W2mef-Rh1HA parasites but not in parental W2mef confirming that the endogenous protein was tagged at the C-terminus (Fig. 2C, left panel). The R513 antibody to the C-terminal region of PfRh1 specifically detected a protein of 120 kDa in W2mef and a doublet of 120/125 kDa in W2mef-Rh1HA. The band at > 150 kDa is a cross-reaction that is consistently seen with this antibody (Figs 2C and 1B). W2mef has three copies of the *PfRh1* gene (Triglia *et al*., 2005) and this is consistent with one copy being HA-tagged while the other two copies remained untagged. These results also indicated that while full-length PfRh1 was HA-tagged it is processed into a 125 kDa product that, as expected, corresponds to the C-terminal region of the protein (Fig. 2C). Additionally, we constructed a W2mef parasite line that expressed a His_6_-tagged PfRh4 protein. To confirm this we used immunoblots with anti-PfRh4 antibodies and detected a 180/190 kDa doublet in W2mefΔ175 parasites and a slightly larger doublet at ∼183/193 kDa in W2mef-Rh4His parasites consistent with the addition of six amino acids at the C-terminus of the protein (Fig. 2D). Tagging of PfRh4 with His_6_ was confirmed using anti-His antibodies that detected a doublet at ∼183/193 kDa in W2mef-Rh4His but not in W2mefΔ175 schizonts (Fig. 2D). Finally, for the tagged EBA-175 protein, anti-His antibodies specifically detected a protein at ∼190 kDa in W2mef-175His parasites but not in the W2mef parent (Fig. 2E), indicating that EBA-175 had been tagged at its C-terminus. This provided parasite lines that expressed the proteins PfRh1, PfRh4 and EBA-175 with epitope tags at the C-terminus.

To ensure that the tagged EBA-175, PfRh4 and PfRh1 proteins had retained their function the ability to invade normal and neuraminidase-treated erythrocytes was determined (Fig. 2F). W2mef is known to be reliant on sialic acid containing receptors for invasion (Triglia *et al*., 2005), a finding that has been corroborated here where there is little invasion into neuraminidase-treated erythrocytes (Fig. 2F). In contrast, W2mefΔ175 parasites have switched to a sialic acid-independent invasion pathway reliant on PfRh4 function (Fig. 2F) (Stubbs *et al*., 2005) and show a similar invasion level into both neuraminidase-treated and untreated erythrocytes. W2mef175His parasites also showed low-level invasion of neuraminidase-treated erythrocytes as seen for the W2mef parent, indicating that they had not switched to PfRh4 and that the His-tagging did not result in loss of function of the EBA-175 protein (Fig. 2F). Invasion of W2mef-Rh1HA parasites was indistinguishable from that of the W2mef parent, suggesting that the HA tagging of PfRh1 resulted in a functional protein because previously it was observed that disruption of PfRh1 expression in the Tak994 parasite line resulted in increased invasion into neuraminidase-treated erythrocytes (Fig. 2F) (Triglia *et al*., 2005). Additionally, the tagged and endogenous PfRh1 colocalize in schizonts and rings, further indicating that HA-tagged PfRh1 was functional. Finally, the parasites expressing His-tagged PfRh4 show increased invasion of neuraminidase-treated erythrocytes consistent with activation of function (Fig. 2F) (Stubbs *et al*., 2005). These results demonstrate that the tagged EBA-175, PfRh1 and PfRh4 proteins are functional in merozoite invasion.

### PfRh1-240 and -120 products may not form a complex for transport to the apical tip

**Fig. 3:**
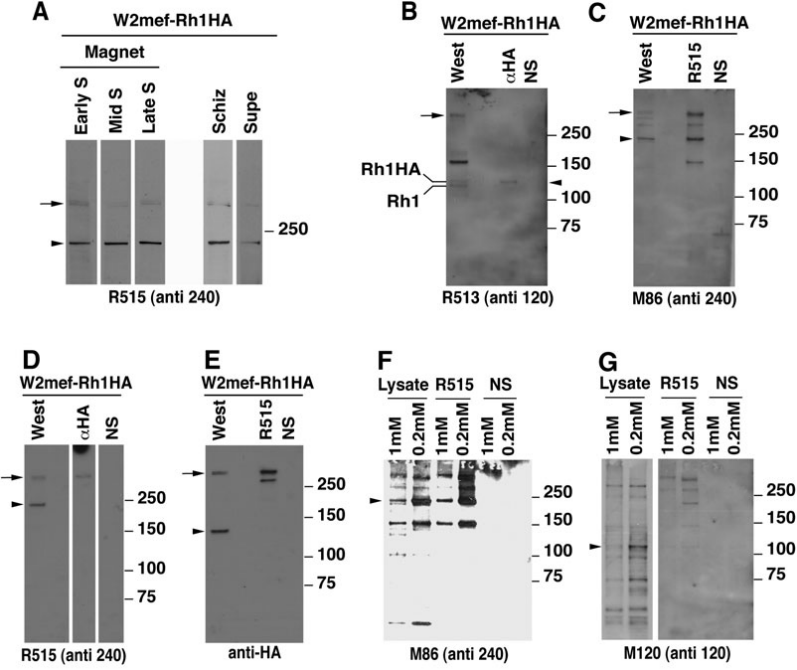
Schizont-processed PfRh1-240 and -120 products most likely do not form a complex for transport to the apical tip. A. Synchronized W2mef-Rh1HA parasites were magnet-purified and samples taken to obtain early, mid or late schizont stages free of rings. For comparison, non-magnet-purified schizonts and culture supernatants were also obtained. Proteins were probed with the anti-Rh1-240 antibody (R515). Unprocessed PfRh1 is denoted by the arrow while the 240 kDa processed product is denoted by the arrowhead. Synchronized W2mef-Rh1HA schizont proteins were either immunoprecipitated with antibodies to Rh1-120 (αHA), antibodies to Rh1-240 (R515) or with normal serum (NS). Immunoprecipitated proteins (labelled with the immunoprecipitating antibody) or W2mef-Rh1HA lysate (labelled West for Western blot) was separated by SDS-PAGE then probed with either (B) R513, (C) mouse 86, (D) R515 or (E) rat anti-HA as shown. The protein detected in the lysate or specifically immunoprecipitated is denoted by an arrowhead, while unprocessed full-length Rh1 at ∼360 kDa is denoted by an arrow. F. The Rh1-240 product can be immunoprecipitated from DSP cross-linked W2mef parasites. M86 against the Rh1-240 product was used in Western blots to detect immunoprecipitated proteins. The Rh1-240 product detected by the mouse antibody in both cross-linked lysates and R515 (anti-240) immunoprecipitates, is denoted by the arrowhead. G. The Rh1-120 product is not immunoprecipitated with antibodies to the 240 kDa Rh1 product. Mouse anti-120 antibodies detect a 120 kDa product in cross-linked lysate (denoted by the arrowhead) but not in material immunoprecipitated with antibodies to the 240 kDa product.

PfRh1 is processed into a 240 kDa N-terminal region and a transmembrane-containing 120 kDa product, and it has previously been shown that the 240 kDa product binds erythrocytes (Triglia *et al*., 2005) and is located at the merozoite apical tip (Taylor *et al*., 2002). In order to determine if the 240 and 120 kDa products are associated as a complex in schizont stages during trafficking to the apical surface and binding to host erythrocytes we prepared proteins from W2mef-Rh1HA schizonts and tested if they could be co-precipitated using specific antibodies (Fig. 3). First, we confirmed that W2mef-Rh1HA schizonts had processed PfRh1 in an identical manner as for the W2mef parent (Fig. 1D). To do this we used synchronized late-stage W2mef-Rh1HA parasites and removed uninfected erythrocytes and ring stages using magnet purification. We then further cultured the parasites to obtain early, mid and late schizont stages, from which proteins were obtained. Proteins were probed with anti-PfRh1-240 antibodies (R515), which confirmed that a very small proportion of unprocessed PfRh1 was present as observed in W2mef (Fig. 3A). Schizonts of W2mef-Rh1HA purified by other methods gave identical results (Fig. 3A). In addition, unprocessed PfRh1 was essentially undetectable in culture supernatant from W2mef-Rh1HA parasites, as was the case for the W2mef parent (Fig. 1D). These results confirmed that processing of PfRh1 in W2mef-Rh1HA parasites was identical to that observed for the W2mef parent.

First we performed immunoprecipitation with anti-HA antibodies and detected PfRh1-120 with anti-PfRh1-120 antibodies (R513) in W2mef-Rh1HA parasites, that expressed a HA-tagged PfRh1 protein. This confirmed that the 125 kDa HA-tagged protein could be specifically immunoprecipitated (arrowhead, Fig. 3B). Second, we showed that the PfRh1-240 protein could be specifically immunoprecipitated with anti-PfRh1-240 (R515) antibodies and detected using antibodies to a second region of the protein (M86) (arrowhead, Fig. 3C). To determine if PfRh1-240 and PfRh1-120 were present in a complex, immunoprecipitation was performed with anti-HA antibodies to pull down the PfRh1-120 fragment and an immunoblot performed with R515 (anti-PfRh1-240) (Fig. 3D). While unprocessed PfRh1 could be detected (arrow, Fig. 3D) with anti-HA antibodies, the 240 kDa fragment was not detectable. This potential lack of association in a complex was confirmed by the reverse experiment in which the PfRh1-240 fragment was immunoprecipitated with R515 antibodies and PfRh1-120 detected using anti-HA antibodies (Fig. 3E). Once again, while unprocessed PfRh1 was detected (arrow, Fig. 3E) PfRh1-120 could not be detected. These results are consistent with the N- and C-terminal processed products of PfRh1 not being present as a complex post cleavage.

It was possible that the putative PfRh1-240/PfRh1-120 complex had been disrupted under the solubilization conditions used for immunoprecipitation and we therefore used a protein cross-linking reagent to provide additional evidence (Fig. 3F and G). Purified W2mef schizonts were incubated with cross-linker dithiobis[succinimidylpropionate] (DSP) and immunoprecipitated with R515 (anti-PfRh1-240) followed by detection of the same fragment with M86 (anti-PfRh1-240) antibodies as a control to confirm that PfRh1-240 could be detected under the cross-linking conditions used (Fig. 3F). The same immunoprecipitates were blotted then probed with anti-PfRh1-120 antibodies (Fig. 3G). Although the PfRh1-120 fragment was readily detected in lysates (Fig. 3G, left panel arrowhead) it was not observed in immunoprecipitations with R515 antibodies. In contrast, the full-length PfRh1 protein was detected. These results are consistent with the PfRh1-240 and PfRh1-120 processed products not forming a complex after cleavage in schizonts.

### Schizont-processed PfRh1-240 colocalizes with PfRh1-120 at the apical tip and tight junction of the merozoite

**Fig. 4:**
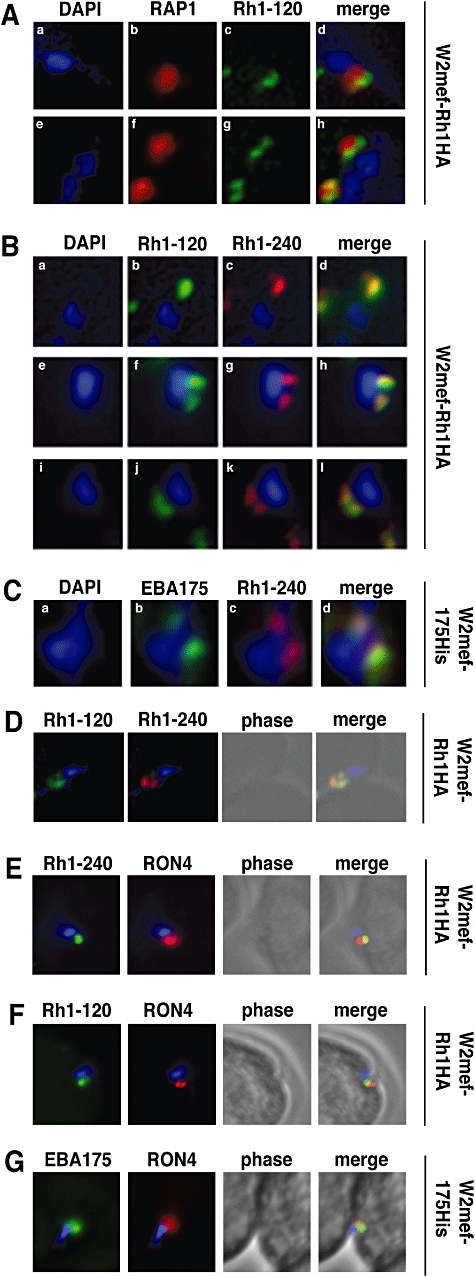
Schizont-processed Rh1-240 colocalizes with Rh1-120 at the apical tip and tight junction of the merozoite. A. The Rh1-120 protein is located at the apical tip and tight junction of merozoites. Free merozoites from the W2mef-Rh1HA line were dual stained with a McAb to RAP1 in the body of the rhoptry and with rat anti-HA antibodies to the tagged 120 kDa Rh1. Anti-HA staining is seen either apical to the RAP1 staining (top panels) or at both sides but posterior to RAP1 staining indicating tight junction staining (bottom panels). Nuclei were stained with DAPI. B. The Rh1-120 protein colocalizes with the Rh1-240 protein at the apical tip and tight junction of merozoites. Free merozoites from the W2mef-Rh1HA line were dual-stained with rat anti-HA antibodies to the tagged 120 kDa Rh1 and with a rabbit antibody (R515) to the 240 kDa Rh1. Colocalization is seen at the apical tip (panels a–d) and at the tight junction (panels e–l) of individual merozoites. C. The Rh1-240 protein colocalizes with EBA-175 at the tight junction of merozoites. Free merozoites from the W2mef-175His line were dual-stained with mouse anti-His antibodies to tagged EBA-175 and with a rabbit antibody (R515) to the 240 kDa Rh1. EBA-175 and Rh1-240 appear to colocalize at the tight junction. D. Free merozoites from the W2mef-Rh1HA line were dual-stained with rat anti-HA antibodies to the tagged 120 kDa Rh1 and with a rabbit antibody (R515) to the 240 kDa Rh1. Simultaneous staining of both the apical tip and the tight junction of a merozoite is seen. E–G. Merozoites of the W2mef-Rh1HA or the W2mef-175His lines were trapped during invasion using cytochalasin D. Rh1-240 was stained with R515, Rh1-120 with rat anti-HA and EBA-175 with mouse anti-His antibodies. PfRON4 was stained either with a mouse antibody (E) or with a rabbit antibody (F and G).

While PfRh1-240 and PfRh1-120 may not form a complex for transport to the apical tip, it was of interest to determine if they colocalized in free merozoites and during erythrocyte invasion (Fig. 4). As we, for the most part, used W2mef-Rh1HA parasites for colocalization experiments, it was important to note that in this tagged parasite, there is only a small proportion of unprocessed PfRh1, with the majority processed to the 240 and 120 kDa fragments (Fig. 3A). In W2mef-Rh1HA parasites PfRh1-120 detected with anti-HA antibodies is located at the apical end of merozoites and contrasts with the rhoptry body marker RAP1 (Fig. 4A, panels a–d). Additionally, we observed a proportion of free merozoites that had established a tight junction, as described previously, allowing localization of EBA-175 in this interaction between the erythrocyte and invading merozoite (O'Donnell *et al*., 2006). In these cases, PfRh1-120 showed a fluorescence pattern consistent with localization at the tight junction with a typical lobed pattern across the merozoite that was generally posterior to RAP1, which remains in the body of the rhoptries (Fig. 4A, panels e–h). This suggested that PfRh1-120 is located at the apical end of the merozoite and moves to the posterior with the tight junction during invasion.

To determine if the PfRh1-120 and PfRh1-240 processed products of PfRh1 colocalize during merozoite invasion we used specific antibodies for detection in immunofluorescence experiments. Many of the merozoites showed a single dot of fluorescence for both PfRh1-120 and PfRh1-240 at the apical end consistent with localization in the neck of the rhoptries (Fig. 4B, panels a–d) as shown previously for the localization of EBA-175 (O'Donnell *et al*., 2006). We also observed some merozoites in which it appeared that a tight junction had been established and moved towards the posterior of the cell. In these cases we observed a lobed pattern typical of the tight junction with both PfRh1-120 and PfRh1-240 showing colocalization (Fig. 4B, panels e–l). This suggests that the processed products of PfRh1 are localized to the apical end of the merozoite and move with the tight junction during invasion. To provide additional data in support of this we did colocalization experiments between EBA-175, an invasion protein that has been localized to the merozoite tight junction, and PfRh1-240 (Fig. 4C, panels a–d) (O'Donnell *et al*., 2006). We observed merozoites in which an apparent tight junction had been established and both EBA-175 and PfRh1-240 were colocalized and had moved towards the posterior of the merozoite. While the panels in Fig. 4A–C have colocalized Rh1-240 with Rh1-120 either at the apical tip or at the tight junction, we also observe many merozoites where there is simultaneous staining of both the apical tip and the tight junction (Fig. 4D). We postulate that in most merozoites, the majority of PfRh1 remains attached at the apical tip during invasion, and these apical tip-derived proteins are also carried into the Ring following erythrocyte invasion.

To provide further evidence that PfRh1-240 and PfRh1-120 are located at the tight junction during merozoite invasion we treated invading cells with cytochalasin D and determined colocalization with RON4 (Baum *et al*., 2008). The RON4 protein is located at the tight junction in invading tachyzoites of *T. gondii* (Lebrun *et al*., 2005) and its orthologue appears to be associated with this transient structure during merozoite invasion in *P. falciparum*. Cytochalasin D is a cell permeable inhibitor of actin polymerization and disrupts formation of actin microfilaments and consequently interferes with invasion, trapping merozoites at the erythrocyte membrane and enriching for those in the process of invading. Merozoites that appear to be in the process of invading erythrocytes show partial colocalization of both PfRh1-120 and PfRh1-240 with RON4 (Fig. 4E-G). Taken together these data suggest that PfRh1-240 and PfRh1-120 colocalize with EBA-175 at the apical end of the parasite at tight junction formation and move towards the posterior end during invasion.

### PfRh1-240 is further processed following invasion

**Fig. 5:**
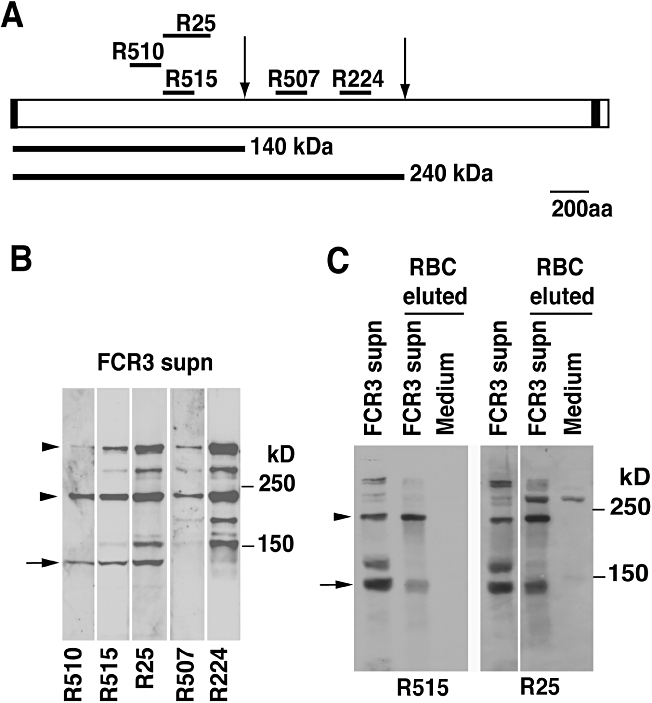
PfRh1-240 is further processed following invasion. A. Schematic representation of the antibodies used to determine the location of the 240 kDa to 140 kDa processing event. B. Culture supernatant from the FCR3 parasite was probed with five antibodies to the 240 kDa product. Each antibody's reactivity with the ∼360 kDa unprocessed Rh1 (arrowhead) and with the 240 kDa (arrowhead) or 140 kDa (arrow) products is shown. C. Culture medium alone or culture supernatant from the FCR3 parasite (FCR3 supn) was bound to erythrocytes, eluted with salt, separated by SDS-PAGE, blotted and probed with antibodies reactive to both the 240 and 140 kDa products. The arrowhead and arrow indicate the reactivity at 240 and 140 kDa respectively.

Since PfRh1-240 binds to the erythrocyte surface and appears to be localized to the tight junction, it was likely that this protein could be further processed during invasion. We analysed proteins from FCR3 culture supernatants with five antibodies to different regions of PfRh1-240 (Fig. 5A). The FCR3 strain has more than 10 copies of the *PfRh1* gene consequently there is a much greater level of this protein compared with other strains and the amount of unprocessed PfRh1 is at higher levels compared with other parasites. The five antibodies detected the full-length protein as well as a major 240 kDa product (Fig. 5B, arrowheads) but the three more N-terminal antibodies (R510, R515, R25) also detected a protein of 140 kDa, suggesting an additional processing event during invasion located between the regions of PfRh1 defined by the antibodies R25 and R507 (Fig. 5B). Binding of FCR3 supernatants to erythrocytes (Haynes *et al*., 1988) confirmed that the 240 kDa product bound red blood cells as has been shown previously and that the 140 kDa subfragment also bound to the host cell (Fig. 5C). This is consistent with a recent study that has defined the erythrocyte-binding region to a smaller region towards the N-terminus of the protein (Gao *et al*., 2008) a domain that is contained within the 140 kDa fragment described here. Our results also show that the 140 kDa fragment generated from the PfRh1-240 protein by further processing is released into the supernatant following invasion.

### PfRh1 and PfRh4 are C-terminally processed at invasion

**Fig. 6:**
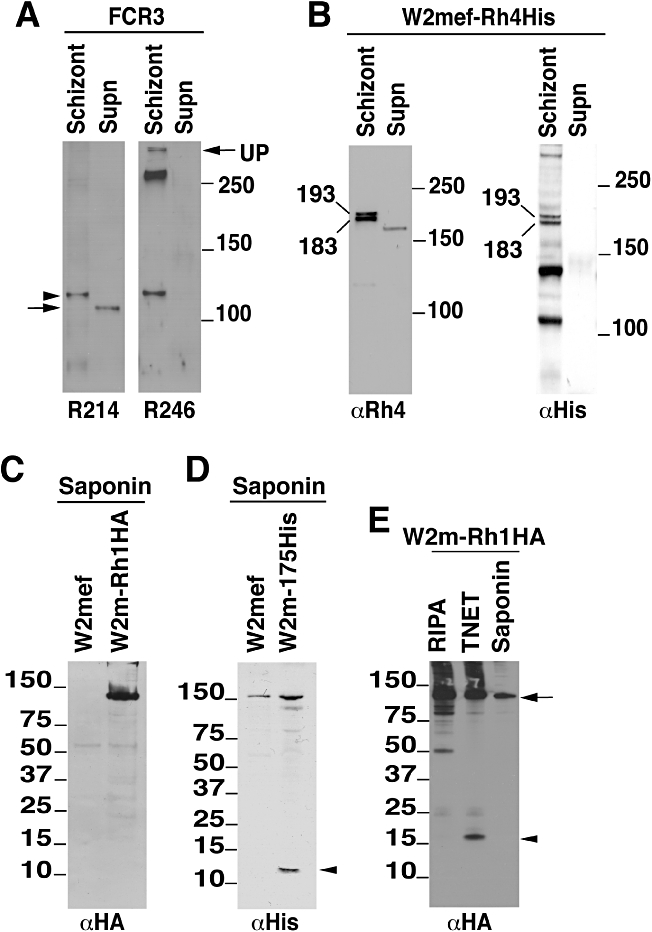
Both PfRh1 and PfRh4 are C-terminally processed at invasion. A. The PfRh1-120 product is C-terminally cleaved. Schizonts or culture supernatant from FCR3 parasites were probed with either R214 (against Rh1-120 but N-terminal to the transmembrane domain) or R246 (Rh1 cytoplasmic tail antibody). The uncleaved 120 kDa product is denoted by an arrowhead while the cleaved product in supernatant is denoted by the arrow. The unprocessed PfRh1 protein detected by R246 is denoted by an arrow and the letters ‘UP’. The band at 250 kDa is an erythrocyte protein cross-reaction that is detected by this antibody. The amount of unprocessed PfRh1 protein detected varies between different antibodies with R214 barely detecting unprocessed PfRh1 while R246 does detect it. B. PfRh4 is C-terminally cleaved. Schizonts or culture supernatant from W2mef-Rh4His parasites were probed either with an anti-Rh4 antibody or with anti-His antibodies. The unprocessed Rh4 products are seen in schizonts at ∼183 and 193 kDa with both antibodies but C-terminal processing has resulted in the loss of detection of these products with anti-His antibodies. C. The HA-tagged C-terminally cleaved PfRh1 stub expected to be ∼14 kDa is not detected by anti-HA antibodies in W2mef-Rh1HA late schizonts containing ∼20% rings. D. The His-tagged, rhomboid cleaved EBA-175 stub expected to be ∼12 kDa is detected by anti-His antibodies (denoted by the arrowhead). E. The HA tagged C-terminally cleaved PfRh1 stub is detected with HRP-labelled anti-HA antibodies. Late schizonts/ring stage cultures of the W2mef-Rh1HA parasite were lysed in either RIPA [1% deoxycholate, 1% TX100, 0.1% SDS, 10 mM Tris (pH 8), 150 mM NaCl], TNET or saponin and soluble proteins immunoprecipitated with rat anti-HA antibodies. Immunoprecipitates were separated by SDS-PAGE and probed with HRP-labelled anti-HA antibodies. Unprocessed Rh1-120 was detected under all three lysis conditions (arrow) while the ∼16 kDa stub was only detected when parasites were lysed in TNET (arrowhead).

The invasion ligand EBA-175 is cleaved within the transmembrane region by a rhomboid protease PfROM4 during merozoite invasion. PfRh1-120 colocalizes with EBA-175 during merozoite invasion and it is likely that this transmembrane-containing protein is similarly processed by a rhomboid protease (O'Donnell *et al*., 2006). Indeed, a portion of PfRh1, including the transmembrane region, has been expressed in COS cells and shown to be cleaved by PfROM4 (Baker *et al*., 2006). In order to test if the endogenous PfRh1-120 fragment is cleaved at the transmembrane region in *P. falciparum*, proteins from schizonts and supernatants were probed with R214 antibodies detecting the 120 kDa C-terminal ectodomain and R246 that detects the cytoplasmic tail (Figs 1A and 6A). In FCR3, R246 antibodies identified a protein of 120 kDa and the full-length PfRh1 protein in schizonts but no bands were observed in supernatants suggesting that the cytoplasmic tail was removed during its release (Fig. 6A). In contrast, while R214 identified the expected 120 kDa protein in schizonts a 110 kDa protein was present in the supernatant consistent with an approximately 10 kDa portion being cleaved from the C-terminus of PfRh1-120, which includes the cytoplasmic tail, resulting in shedding of the 110 kDa ectodomain into the supernatant.

To determine if other members of the PfRh family of proteins are also processed at the transmembrane region consistent with the function of a rhomboid protease we used W2mef-Rh4His parasites that express a His_6_-tagged PfRh4 protein (Fig. 2). In W2mef-Rh4His parasites, antibodies to PfRh4 detect a doublet of ∼183/193 kDa in schizonts and a protein of ∼160 kDa in supernatant (Fig. 6B). While this showed that processing of PfRh4 had occurred, it did not prove that the protein was cleaved at the C-terminus. In order to determine if C-terminal processing had occurred, anti-His antibodies were used and the expected doublet at ∼183/193 kDa was observed in schizonts but the 160 kDa band was not present in supernatant (Fig. 6B). This indicated that the tagged PfRh4 protein was C-terminally cleaved removing approximately 20 kDa, which corresponds in size to the transmembrane, cytoplasmic tail and His_6_ consistent with the function of a PfROM protease to release the ectodomain into the supernatant.

To further establish that PfRh1-120 was cleaved at the transmembrane region, immunoblot analysis was used to detect the cleaved stub that would be carried into ring stages during merozoite invasion. However, numerous experiments with saponin lysed early ring-stage parasites failed to detect the expected PfRh1 fragment of approximately 13 kDa (Fig. 6C). In contrast, the PfROM4 cleaved stub of EBA-175, which was tagged with His_6_, was detected at the expected size of 12 kDa (Fig. 6D). We hypothesized that failure to detect the PfRh1 cleaved stub may have occurred due to differential solubility in detergents such as saponin. To address this possibility we used late schizonts/rings of the W2mef-Rh1HA parasites, lysed either in RIPA, TNET or saponin, immunoprecipitated with rat anti-HA antibodies, then detected the C-terminus of PfRh1-120 with horseradish peroxidase (HRP)-labelled anti-HA antibodies (Fig. 6E). While the unprocessed HA-tagged PfRh1-120 protein was readily detected using all three detergents, the cleaved stub, which ran with an apparent molecular weight of ∼16 kDa, was only efficiently detected when TNET was used to extract proteins (Fig. 6E). Therefore, PfRh1-120 is C-terminally cleaved *in vivo* during invasion to release the ectodomain into the supernatant and this is consistent with the action of a rhomboid protease such as PfROM4 at the tight junction (Baker *et al*., 2006).

### C-terminally unprocessed PfRh1-120 and schizont-processed PfRh1-240 are carried into the Ring after invasion

**Fig. 7:**
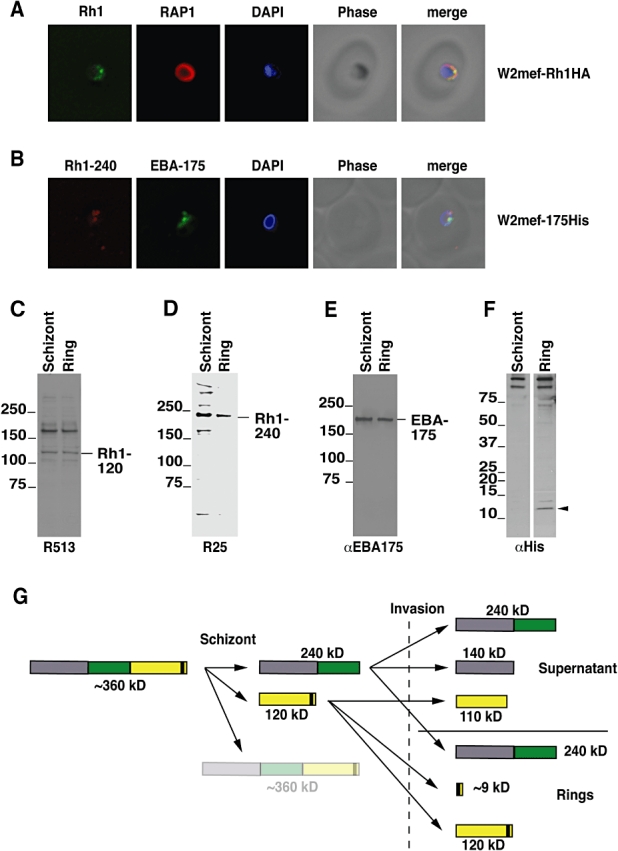
C-terminally unprocessed PfRh1-120 and schizont-processed Rh1-240 are both carried into the Ring. A. W2mef-Rh1HA Rings were dual-stained with mouse anti-RAP1 and rat anti-HA antibodies (to detect the unprocessed Rh1-120 product). B. W2mef-175His Rings were dual-stained with rabbit anti-Rh1-240 (R515) and mouse anti-His antibodies (to detect tagged EBA-175). C–F. Magnet-purified ring-free schizonts and schizont-free Rings of the W2mef-175His line were lysed and the proteins separated by SDS-PAGE. These preparations were smeared after purification and analysed for presence of contaminating stages. In the purified schizont stages no ring stage infected erythrocytes were observed in the smears with 97% schizonts and 3% uninfected erythrocytes and 0% ring stage infected erythrocytes. For the purified ring stage infected erythrocytes in smears they were 98% pure with 2% uninfected erythrocytes and 0% schizonts. Blotted proteins were probed with antibodies to Rh1-120 (C), Rh1-240 (D), EBA-175 (E) or anti-His to the tagged rhomboid-processed EBA-175 stub (F). G. Schematic diagram illustrating the fate of various PfRh1 processed products. The ∼360 kDa unprocessed PfRh1 is present at low levels in schizont stages and represented by the faint outline of the unprocessed product.

To determine the fate of PfRh1 during invasion we used immunofluorescence and immunoblots with ring stage parasites to follow the proteolytically cleaved products. W2mef-Rh1HA ring stage parasites were incubated with anti-HA to detect the C-terminus of PfRh1 with respect to anti-RAP1 antibodies, a marker of the parasitophorous vacuole. PfRh1 fluorescence with the cytoplasmic tail gave a dotty appearance at the ring periphery while RAP1 gave a more uniform staining pattern consistent with a parasitophorous vacuole localization (Fig. 7A). In similar W2mef-175His ring stage parasites, antibodies to the PfRh1-240 region gave a punctate fluorescence at the ring periphery, as did anti-His antibodies to the EBA-175 cytoplasmic tail and these antibodies showed some colocalization between the two proteins (Fig. 7B). This suggests that PfRh1 peptides are carried into invaded rings; however, in these experiments it was not possible to distinguish between the full-length and processed products.

In order to determine which PfRh1 processed products were carried into ring stage-infected erythrocytes in W2mef-175His parasites, we used schizonts in which all ring stages were removed and ring stages in which all schizonts had been removed (see *Experimental procedures*) for immunoblot experiments. Using R513 antibodies we detected the PfRh1-120 product, which includes the transmembrane region and cytoplasmic tail, in both schizont and ring stage parasites indicating that this protein was carried into the newly invaded erythrocyte (Fig. 7C). Similarly, R25 antibodies that detect PfRh1-240 showed that this protein was also carried into the ring stage (Fig. 7D). To determine if unprocessed EBA-175 could also be carried into the ring, we probed an identical blot with anti-EBA-175 antibodies. Once again the result showed that unprocessed EBA-175 was carried into ring stages following merozoite invasion (Fig. 7E) and when the same proteins were separated on a higher percentage gel, the EBA-175 rhomboid-processed stub at ∼12 kDa was also detected in ring but not schizont preparations (Fig. 7F). These results suggest that a proportion of PfRh1-120 and EBA-175 proteins are C-terminally cleaved at invasion reflecting the amount incorporated into the tight junction, while the rest remains unprocessed at the merozoite apical tip allowing it to be carried into the invaded erythrocyte with the newly formed ring stage.

## Discussion

PfRh proteins play a key role in merozoite invasion and this has been shown primarily by gene knockout of specific family members in *P. falciparum* in which the derived parasites, lacking expression of specific ligands, utilize a different pattern of erythrocyte receptors (Dolan *et al*., 1990; Duraisingh *et al*., 2003; Stubbs *et al*., 2005). While it has been demonstrated that some members of the PfRh protein family, such as PfRh1 and PfRh4, can bind specifically to the erythrocyte surface (Rayner *et al*., 2001; Stubbs *et al*., 2005; Triglia *et al*., 2005; Gaur *et al*., 2007; Gao *et al*., 2008; Tham *et al*., 2009) it has not been known if they play a role in the tight junction with the host cell nor any understanding of the fate of the proteins during and after the invasion process. In this study, we have analysed PfRh1 and shown that it undergoes a series of proteolytic cleavage events before and during invasion and have followed these processed products to determine their fate. Our results show that processing of both PfRh1 and PfRh4, and by extrapolation all membrane-bound members of this protein family, is important for function and release of these proteins on the merozoite surface and that they along with EBA-175 (O'Donnell *et al*., 2006) are important components of the tight junction during merozoite invasion.

**Fig. 8:**
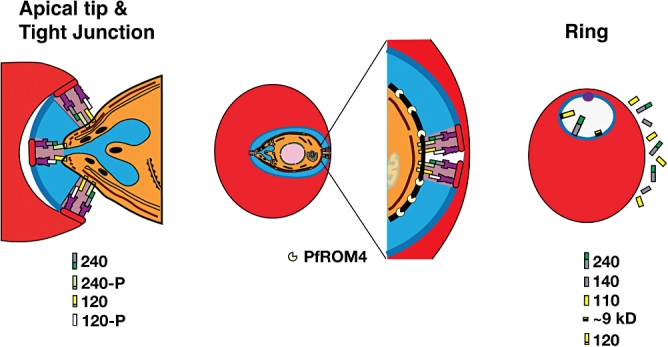
A model for invasion incorporating the roles and ultimate locations of various PfRh1 processed products. Once an erythrocyte has been targeted for invasion, proteins of the EBL and PfRH families, already at the apical tip of the merozoite, begin the invasion process. As PfRh1 is already processed to 120 and 240 kDa products prior to invasion and as both products are at the tight junction, the existence of both a 240 partner protein (240-P) and a 120 partner protein (120-P) must be envisaged. The 240-P protein most likely has a transmembrane domain in order to anchor the Rh1-240 binding domain to the merozoite surface while the 120-P protein most likely binds an erythrocyte receptor and is anchored to the merozoite surface by the Rh1-120 protein. The rhoptry contents together with components of the erythrocyte membrane fuse to form the nascent parasitophorous vacuolar membrane shown in a darker blue colour. While the majority of the Rh1-240 and Rh1-120 proteins remain at the apical tip, a smaller proportion are incorporated into the tight junction, which moves to the posterior of the merozoite by connections to the underlying actin–myosin motor complex. Once at the posterior the components of the tight junction are cleaved by intramembrane rhomboid proteases such as PfROM4. The right panel shows the final location of the various PfRh1 processed products either within the newly developing Ring or in culture supernatant.

We have followed PfRh1 to enable an understanding of the role of proteolytic processing and the fate of the processed fragments during schizont development and subsequent merozoite invasion of the host erythrocyte. In mature schizont stages of *P. falciparum*-infected erythrocytes PfRh1 is expressed as a large protein of 360 kDa and quickly processed post-Golgi into two sections corresponding to the 240 kDa N-terminus and a 120 kDa C-terminal section containing the transmembrane region (Figs 7G and 8). The cleaved 240 and 120 kDa fragments appear not to form a complex although it is difficult to prove this directly. However, the results are consistent with these two fragments not forming a complex which would suggest that PfRh1-240 may bind to an unknown membrane-bound protein allowing it to be escorted to the rhoptry neck where this family of proteins is initially located.

This would be similar to the rhoptry complex proteins where RAP1 escorts RAP2 and RAP3 in *P. falciparum* (Baldi *et al*., 2000) as well as other proteins such as TgMIC6 that serves as an escorter for the soluble adhesins TgMIC1 and TgMIC4 in *T. gondii* (Meissner *et al*., 2002). During merozoite invasion a tight junction is formed with receptors on the erythrocyte membrane in which only a portion of the PfRh1-120 and PfRh1-240 pool participate, and move with the tight junction to the posterior of the parasite (Fig. 8). The remaining PfRh1 processed products not participating in the tight junction, remain at the apical end of the merozoite and play no role in invasion, resulting in their incorporation into the newly invaded ring stage (Fig. 8). The final processing of the PfRh1-240 protein to a 140 kDa erythrocyte binding domain that is shed into the supernatant, may be an event required for disengagement of the ligand–receptor complex that is required during the invasion process (Sibley, 2004).

The PfRh1-120 protein is further processed at the final stages of erythrocyte invasion in a way that is consistent with cleavage by PfROM4 as has been described for EBA-175 (O'Donnell *et al*., 2006). The cleaved 110 kDa fragment is released into the supernatant while the remaining 9 kDa stub consisting of the transmembrane region and cytoplasmic tail is taken into the invaded ring stage and degraded. The transmembrane region of PfRh1 has been shown previously to be cleaved by PfROM4 by expression in COS cells (Baker *et al*., 2006). Production of a 9 kDa PfRh1 membrane-bound fragment is consistent with cleavage by this protease during invasion. Demonstration that PfRh4 was similarly cleaved within the transmembrane region is also consistent with cleavage by PfROM4 and by analogy suggests that other members of the PfRh protein family are processed by this membrane-associated protease during merozoite invasion and that these proteins are associated and play key roles in the tight junction during merozoite invasion.

A recent study of PfRh1 has narrowed down the erythrocyte-binding domain to 40 kDa in the N-terminus by heterologous expression of small regions (Gao *et al*., 2008) and this falls within PfRh1-240 and the 140 kDa processed product that is shed into the supernatant during merozoite invasion. Both of the PfRh1 processed products bind to erythrocytes and contain the 40 kDa binding region. Interestingly, in the study identifying the 40 kDa binding domain of PfRh1 they used an antibody to a region of the protein that would fall within the 120 kDa processed product, yet it detected a 240 kDa product which would correspond to the N-terminal processed product rather than the C-terminal region as we suggest (Gao *et al*., 2008). In our study, we have used antibodies made to four independent regions of the C-terminal processed fragment all of which detect the PfRh1-120 kDa product. Also we have tagged the PfRh1 protein at the C-terminus and can identify the expected ∼125 kDa product. Interestingly, they also detected a 120 kDa together with the approximately 240 kDa band using the antibody generated; however, they suggest that the 120 kDa band is non-specific cross reactivity while our data indicate that it is indeed the correct PfRh1-120 fragment described here. Antibodies made to PfRh proteins can display major cross-reactivity in immunoblots and this is an important factor in interpreting specific reactivity as well as testing these antibodies for their ability to inhibit invasion (Triglia *et al*., 2005). We have circumvented these problems by generating antibodies to multiple non-overlapping regions of the PfRh1 protein, constructing transgenic parasites expressing a tagged version of the gene and also using a *P. falciparum* strain in which the *PfRh1* gene has been disrupted (Triglia *et al*., 2005).

Formation and movement of the tight junction across the merozoite surface for *P. falciparum* is a rapid process that is difficult to follow experimentally. Previous studies have suggested that the duffy binding protein (PkDBP) in *P. knowlesi* is required for tight junction formation (Singh *et al*., 2005) and presumably the protein engaged with erythrocyte receptors moves with the junction. PfRon4 and AMA1 localize with the tight junction during invasion of *T. gondii* (Lebrun *et al*., 2005) and a similar localization has been found in *P. falciparum* (Alexander *et al*., 2006). There is evidence that EBA-175 in *P. falciparum* is also engaged in the tight junction through its interaction with glycophorin A and shedding of the ectodomain occurs during invasion by cleavage with PfROM4 (O'Donnell *et al*., 2006). Our data are consistent with processed PfRh1 also being associated with the tight junction with the PfRh1-240 protein binding to the erythrocyte via a sialic acid-dependent interaction with a host receptor. It is possible that PfRh1-240 complexes with an unknown membrane protein that may also be cleaved by PfROM4 or alternatively, the cleavage event we have identified that releases a 140 kDa portion of this protein into the supernatant may be the event required for release of the ligand–receptor interaction that is required for invasion. The PfRh1-120 protein is also associated with the tight junction and it is possible that it interacts with a second soluble protein that binds to the erythrocyte (Fig. 8). Irrespective of the exact interaction, both PfRh1-240 and PfRh1-120 are located at the tight junction and must play a key role in its organization. It is likely that the protein complexes within the tight junction are highly organized with distinct domains defined by the presence of specific protein complexes. Determining the protein content and higher order structure of the tight junction will be important to our understanding of merozoite invasion.

The PfRh family of proteins is localized to the apical end of the invading merozoite and their role appears to be initial binding to the erythrocyte (Galinski *et al*., 1992). EBA-175 and its paralogues play a very similar role and we have suggested previously that these protein families are functionally equivalent and act after merozoite reorientation to bind the apical end to the erythrocyte allowing establishment of the tight junction, a complex in which they perform a key function. This is supported by the observation that disruption of the *EBA-175* gene in the W2mef strain of *P. falciparum* results in selection of parasites that have activated a silenced *PfRh4* gene allowing the use of receptors other than glycophorin A (Stubbs *et al*., 2005). This suggests that loss of EBA-175 function is complemented by the expression of PfRh4 consistent with the concept that the ebl and PfRh protein families serve the same function. Understanding the role of the PfRh and ebl proteins in alternate invasion pathways is critical to our ability to devise rational vaccine combinations that will provide immune responses that block a broad array of the pathways and ultimately may disrupt merozoite invasion.

## Experimental procedures

### Parasite cultures

*Plasmodium falciparum* asexual stages were maintained in human O+ erythrocytes. 3D7 is a cloned line derived from NF54 obtained from David Walliker at Edinburgh University. W2mef is a cloned line derived from the Indochina III/CDC strain and FCR3 is a cloned line. Tak994 is from South-east Asia and Tak994ΔRh1 is a cloned line containing a disrupted *PfRH1* gene which has been described previously (Triglia *et al*., 2005).

### Plasmids and transfection

Plasmids for transfection of *P. falciparum* were constructed from pARL (Marti *et al*., 2004) by removing the crt5′ and GFP regions, and replacing with a region encoding either three HA tags (pHA3) or six His residues (p6His). Genomic DNA from the 3D7 parasite extracted from late stages as described (Triglia and Cowman, 1994) was used as template to amplify a ∼850 bp fragment from the C-terminal end of the *PfRh1* gene by polymerase chain reaction (PCR) using the following primers: [5′-AGCTagatctGAATGGAATCAAGAATATAAAC] and [5′-AGCTctgcagCAATATAATCATTATCAAAAAATATC]. A ∼820 bp C-terminal fragment of the *PfRh4* gene was amplified from genomic DNA with the following primers [5′-AGCTagatctAATGAAATGGGATATGGCATAAC] and [5′-AGCTctgcagCTATGTCATTAAAATCTTCATTTTC]. A ∼950 bp PCR fragment from the C-terminal end of the *EBA-175* gene was amplified with the following primers from 3D7 cDNA: [5′-AGCTctgcagCTTTCTTAAAATTAATATCATTTATATCC] and [5′-AGCTggatccGAAGCAAAAATGAAAGGAAAT]. The BglII/BamHI and PstI sites were used to clone all PCR fragments into both pHA3 and p6His transfection plasmids.

Transfection with 80 μg of purified plasmid DNA (Qiagen) and selection for stable transfectants by single recombination cross-over was carried out as described previously (Duraisingh *et al*., 2002).

### Antibodies

Various *PfRh1* fragments for subcloning into the pGEX vector were amplified by PCR from 3D7 gDNA. The fusion protein used to generate the R510 antibody used the primers [5′-GATCggatccAAAAATGATATTACACACAAAG] and [5′-TATCctcgagTCCAGAAATAAGTTGAATCGTCTC], R515: [5′-TCTggatccGAAGATAAACATGAATCCAATCC] and [5′-GATCctcgagGCGAATTTCCTTTTCCTTAGC], R25: [5′-TCTggatccGAAGATAAACATGAATCCAATCC] and [5′-AAATctcgagTTGTAGAAGATCTATTTCGTGTG], R507: [5′-GATggatccGATGAATACCATATAGCCCTTTC] and [5′AGGCctcgagTGTAAGAAGATTTTTTCTGTTTTTTTG], R511: [5′-GATCggatccATACATACATATATCCAAGAA] and [5′GATCctcgagTGTTTGTATATAATTCAAGGT], R513: [5′-GATCggatccGACATCATACAGAAAAAAGAA] and [5′GATCctcgagTGTAATTAGATGATTGTCACA] and R246: [5′-GATCggatccTAATAAACAAGAATATGATAAAGAG] and [5′-GATCctcgagTTAAATATAATCATTATCAAAAAATATC]. Other *PfRh1* fragments subcloned into the pMAL vector, were also amplified by PCR. The fusion protein used to generate the R224 antibody used the primers [5′-GATCggatccAATGATATTACACATATAAAG] and [5′-GATCctcgagGTCTATATGTTTCATTTGTGA], R214: [5′-GATCggatccGAAAGACAAAACGATGTACAC] and [5′-GATCctcgagAAAATAAGTGTCTTCTTTGTT]. Each fusion protein was affinity-purified on either glutathione-agarose (pGEX) or maltose binding protein (pMAL), then used to immunize rabbits and mice. The rabbit anti-Rh1 antibodies were either affinity-purified on the immunizing fusion protein coupled to Sepharose 4B or purified on Protein G-Sepharose then depleted of antibodies to GST or MBP. The mouse anti-Rh1 antibodies were used as serum and not further purified. The anti-His antibodies used were a pentaHis mouse monoclonal (Qiagen) and the anti-HA antibodies used were either the mouse monoclonal 12CA5 or the rat monoclonal 3F10 (Roche Applied Science). The anti-PfRON4 antibodies have been previously described (Richard *et al*., 2009).

### Erythrocyte binding, SDS-PAGE and Immunoblot analysis

Culture supernatants enriched in proteins released during merozoite invasion were obtained by synchronization of parasite cultures followed by treatment with trypsin and neuraminidase in order to prevent reinvasion of erythrocytes following schizont rupture, as described (Triglia *et al*., 2005). Total proteins from schizont stage parasites were obtained by synchronization and further cultured until mature schizonts were present. Parasite proteins were then obtained by saponin lysis of erythrocytes. All parasite preparations were made in the presence of COMPLETE protease inhibitors (Roche). Parasites in Fig. 7C–F were obtained by synchronization and further culture until there were ∼80% schizonts and ∼20% newly invaded rings. The culture was then separated into ring-free schizonts and schizont-free rings by magnetic separation (CS columns, Miltenyi Biotec) and parasite proteins obtained as above.

Proteins were separated on either 6% Laemmli or 3–8% Tris Acetate (for large proteins) or 4–12% Bis Tris with MES buffer (for small proteins) SDS-PAGE gels (Invitrogen). Western blotting onto nitrocellulose (0.45 μm, Schleicher and Schuell) was performed according to standard protocols and blots were processed with a chemiluminescence system (ECL, Amersham). Erythrocyte binding assays using enriched culture supernatant from FCR3 parasites were performed as described previously (Triglia *et al*., 2001). Bound proteins were separated on SDS-PAGE gels, Western blotted, then probed with anti-PfRh1 antibodies.

### Erythrocyte invasion assay

Uninfected erythrocytes were treated with enzymes as described previously (Triglia *et al*., 2005). The invasion assay was carried out essentially as previously described (Reed *et al*., 2000). Erythrocytes infected with ring stages were synchronized twice followed by pretreatment with high trypsin and neuraminidase to prevent reinvasion into these erythrocytes. Experiments were performed with the addition of new erythrocytes, either untreated or treated with neuraminidase (66.7 mU ml^−1^). The experiments in Fig. 1C were performed in triplicate in round-bottomed microtitre plates. Enzyme-treated or untreated erythrocytes at 4% haematocrit were inoculated with infected erythrocytes to give a final parasitaemia of 1.0% and haematocrit of 4% in a total volume of 100 ml per well. Forty-eight hours later following reinvasion, wells were smeared, Giemsa stained and newly invaded rings counted. The ratio of invasion into neuraminidase-treated cells versus invasion into untreated cells was determined.

### Immunoprecipitation and cross-linking

Saponin-lysed W2mef-Rh1HA schizonts were solubilized in TNET (1% TX100, 150 mM NaCl, 10 mM EDTA, 50 mM Tris pH 7.4) and immunoprecipitated either with rat anti-HA, rabbit anti-Rh1-240 (R515) antibodies or normal serum as shown in Fig. 3B–F. Washed immunoprecipitates were separated by SDS-PAGE, blotted to nitrocellulose, then probed with antibodies of a different species to the immunoprecipitating antibodies, to avoid reactivity with immunoglobulin heavy and light chains. For cross-linking experiments in Fig. 3F and G, synchronized W2mef parasites at the trophozoite stage were magnet-purified as before to remove uninfected erythrocytes and further cultured to mature schizonts. Parasites were washed free of amines and the cross-linking agent DSP (Pierce) added for 30 min. Cross-linked parasite proteins were solubilized in TNET, then immunoprecipitated as above with rabbit anti-240 PfRh1 antibodies. Washed immunoprecipitates were reduced in β-mercaptoethanol to reverse the cross-linking by thiol cleavage before separation by SDS-PAGE, blotted to nitrocellulose, then probed with mouse antibodies.

### Microscopy and Immunofluorescence

Light microscopy was performed with synchronized parasites at schizont, merozoite and ring stages. Schizont and ring stages were fixed in solution with 4% paraformaldehyde and 0.0075% glutaraldehyde (ProSciTech) as described (Tonkin *et al*., 2004). Merozoites were obtained by air-drying smears of late-stage parasites, then fixing either in 100% methanol at −20°C or on the slide with paraformaldehyde/glutaraldehyde as above. In some experiments, merozoite invasion was arrested using 0.1 μM cytochalasin D (Baum *et al*., 2008). For dual-colour fluorescence, fixed parasites in solution or on slides were blocked in 3% BSA in PBS, before both primary antibodies were added. Parasites were washed, secondary Alexa Fluor 488/594 antibodies added, then mounted with VectaShield (Vector Laboratories) containing 1 μg ml^−1^ DAPI. Images were captured with a Carl Zeiss Axioscop2 microscope with an AxioCam camera and Axiovision 3 software. Single z-stacks shown were processed with AxioVision deconvolution software.
